# Supplementary material for: Spatial profiling identifies regionally distinct microenvironments and targetable immunosuppressive mechanisms in pediatric osteosarcoma pulmonary metastases
Source: bioRxiv. 2025 Jan 24:2025.01.22.631350. Preprint. [Version 1] doi: 10.1101/2025.01.22.631350 (PMC11785069; doi:10.1101/2025.01.22.631350)
Supplement: Supplement 1 [file media-1.pdf]

Long Name	Short Name	Genes
Adventitial Fibroblasts	Adventitial_Fibro	COL6A2, SFRP2, IGFBP6, MMP2
Airway Smooth Muscle Cells	ASMC	DES, ACTA2, LGR6, DSTN, TPM2, TAGLN, MYH11, ACTG2, MYLK
Alveolar Epithelium Type 1	AT1	HOPX, AGER, RTKN2, EMP2, CLDN18, LMO7, CLIC3, KRT7
Alveolar Epithelium Type 2	AT2	SFTPC, LAMP3, SLC34A2, SFTPB, SFTPA1, NPC2, NAPSA
Alveolar Fibroblasts type 1	Alv_Fibro1	TCF21, WNT2, SLC38A5, MEOX2, OLML3, VCAM1, COL13A1, ENPEP, ADH1B, G0S2, LBH, ITGA8, CDH11, PLXDC2, CDO1
Alveolar Fibroblasts type 2	Alv_Fibro2	MFAP5, SCARA5, COL14A1, GPC3, CCDC80, RARRES2, LGALS1, PCOLCE, OGN, FSTL1
Alveolar Macrophages	Alv_Mac	ALOX5AP, CD68, CTSD, FCER1G, MARCO, SPI1, SIGLEC1, ABCG1, FABP4
Arterial Endothelial Cells	AEC	DKK2, GJA5, SERPINE2, HEY1, EFNB2, NOTCH1, BMX, CXCL12
B Cells	B_cells	CD69, CORO1A, LIMD2, BANK1, LAPTM5, CXCR4, LTB, CD79A, CD37, MS4A1
Basal Cells	Basal	KRT5, TP63, KRT14, NGFR, ITGA6, IGFBP4, ALCAM
Capillary Endothelial Cells Type 1	CAP1	APLNR, IL7R, GPIHBP1, FCN3, EDN1, SLC6A4, TEK
Capillary Endothelial Cells Type 2	CAP2	CA4, APLN, EDNRB, HPGD
CD4 T-cells	CD4_T	CORO1A, KLRB1, CD3E, LTB, CXCR4, IL7R, TRAC, IL32, CD2, CD3D
CD8 T-cells	CD8_T	CD8A, CD3E, CCL4, CD2, CXCR4, GZMA, NKG7, IL32, CD3D, CCL5
Ciliated Airway Epithelium	Ciliated	FOXJ1, RSPH1, DYBLRB2, FAM183A, NME5, TPPP3, TUBA1A, TUBB4B, TMEM190
Classical Monocytes	Class_Mono	LST1, IL1B, LYZ, COTL1, S100A9, VCAN, S100A8, S100A12, AIF1, FCN1
Dendritic Cells	DC	CORO1A, MS4A6A, ITGB2, GPR183, HLA-DRB1, HLA-DPB1, HLA-DPA1, HLA-DQB1, HLA-DQA1, HLA-DMA
Fibromyocytes	Fibromyo	NEXN, ACTG2, LMOD1, PPP1R14A, DES, FLNA, TPM2, PLN, SELM
Inflammatory Monocytes	Infl_Mono	S100A8, S100A9, CD14, VCAN
Interstitial Macrophages	Int_Mac	C1QA, C1QB, C1QC, IL1B, MS4A4A, C3AR1, CD163, FCGR2A, NPL, SLCO2B1
Ionocytes	Ionocyte	CFTR, FOXI1, ASCL3, ATP6V0B, AZGP1, HES6, TMEM61
Lymphatic Endothelial Cells	LEC	PROX1, MMRN1, CCL21, PDPN, PTX3, NRP2, NRF2, FOXC2
Mesothelial Cells	Meso	WT1, FREM2, UPK3B
Monocyte-derived Macrophages	Mono_Mac	LYZ, ACP5, TYROBP, LGALS1, CD68, AIF1, CTSL, EMP3, FCER1G, LAPTM5
Myoepithelial Cells	MEC	KRT14, MYH11, TP63, KRT5, ACTA2, TAGLN
Myofibroblasts	Myofibro	CALD1, CYR61, TAGLN, MT1X, PRELP, TPM2, GPX3, CTGF, SPARCL1
Neutrophils	Neut	S100A8, S100A9, IFITM2, FCGR3B, IL1B, CCR2, CSF1R
NK Cells	NK	GZMA, CD7, CCL4, CST7, NKG7, GNLY, CTSW, CCL5, GZMB, PRF1
Non-Classical Monocytes	NonClass_Mono	PSAP, FCGR3A, FCN1, CORO1A, COTL1, FCER1G, LAPTM5, CTSS, AIF1, LST1
Osteoclasts	Osteoclast	ACP5, CTSK, ITGB3, MMP13, MMP9
Osteosarcoma Signature	Osteosarcoma	COL9A1, RUNX2, IBSP, MRC2, COL5A3, VIM, SPP1, MIK67, COL2A1, COL12A1, ALPL, LOX, TOP2A, COL11A1, COL11A2, PDGFD, CTHRC1, CLEC11A, TNC, COL27A1, SOX9, TGFBI, SATB2
Patrolling Monocytes	Patrolling_Mon	CDKN1C, PTP4A3, HES4, TNFRSF8
Pericytes	Pericyte	LAMC3, TRPC6, HIGD1B, PDGFRB, COX4I2, KCNK3, NOTCH3, CSPG4,
Plasma Cells	Plasma	ITM2C, TNFRSF17, FKBP11, IGKC, IGHA1, IGHG1, CD79A, JCHAIN, MZB1, ISG20
Plasmacytoid Dendritic Cells	pDC	CLEC4C, LILRA4, IRF7, PLD4
Regulatory T-cells	Treg	FOXP3, CTLA4, IL2RA
Serous Cells	Serous	LTF, LYZ, CCL28, SAA2, PIGR, SLPI
Smooth Muscle Cells	SMC	PRKCDBP, NDUFA4L2, MYL9, ACTA2, MGP, CALD1, TPM1, TAGLN, IGFBP7, TPM2
Subpleural Fibroblasts	Subpleural_Fibro	SERPING1, C1R, NNMT, MT1E, MT1X, PLA2G2A, SELM, MT1M
Systemic Venous Endothelial Cells	SVEC	ACKR1, COL15A1, ABCB1, VWA1
Vascular Endothelial Cells	VEC	ACKR1, PRDD23, VWF, CLU, EPHB4, IGFBP7, NR2F2
