# Supplementary material for: Spatial profiling identifies regionally distinct microenvironments and targetable immunosuppressive mechanisms in pediatric osteosarcoma pulmonary metastases
Source: bioRxiv. 2025 Jan 24:2025.01.22.631350. Preprint. [Version 1] doi: 10.1101/2025.01.22.631350 (PMC11785069; doi:10.1101/2025.01.22.631350)
Supplement: Supplement 2 [file media-2.pdf]

Target	Tag	Clone	Dilution	Catalog ID	Vendor
A-SMA	141Pr	1A4	600	3141017D	SBT
Vimentin	143Nd	D21H3	300	3143027D	SBT
CD68	159Tb	KP1	300	3159035D	SBT
CD8a	162Dy	D8A8Y	200	3162035D	SBT

Supplemental Table 2
