# Supplementary figures and images for: Spatial profiling identifies regionally distinct microenvironments and targetable immunosuppressive mechanisms in pediatric osteosarcoma pulmonary metastases
Source: bioRxiv. 2025 Jan 24:2025.01.22.631350. Preprint. [Version 1] doi: 10.1101/2025.01.22.631350 (PMC11785069; doi:10.1101/2025.01.22.631350)

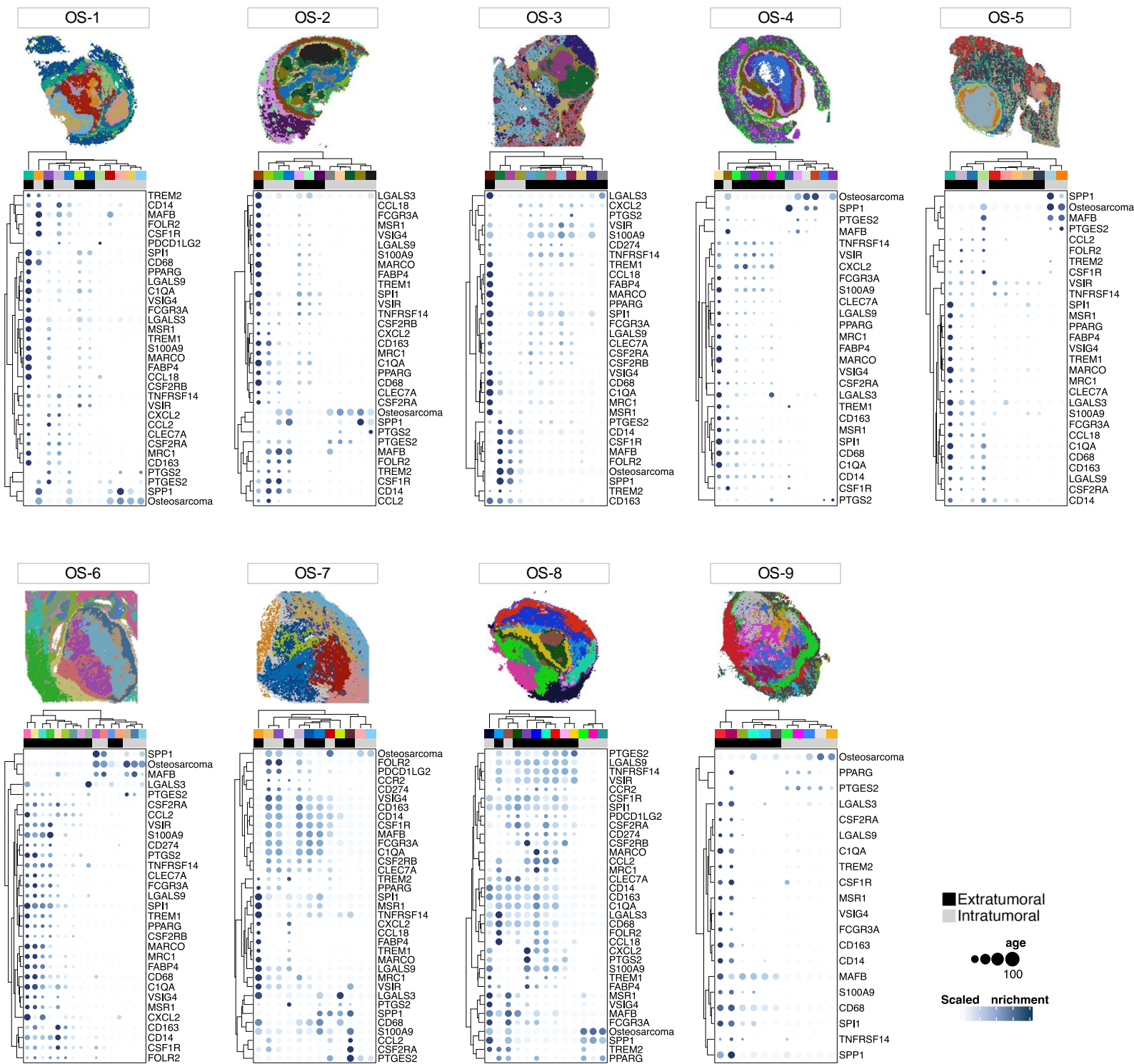

Supplemental Figure 1

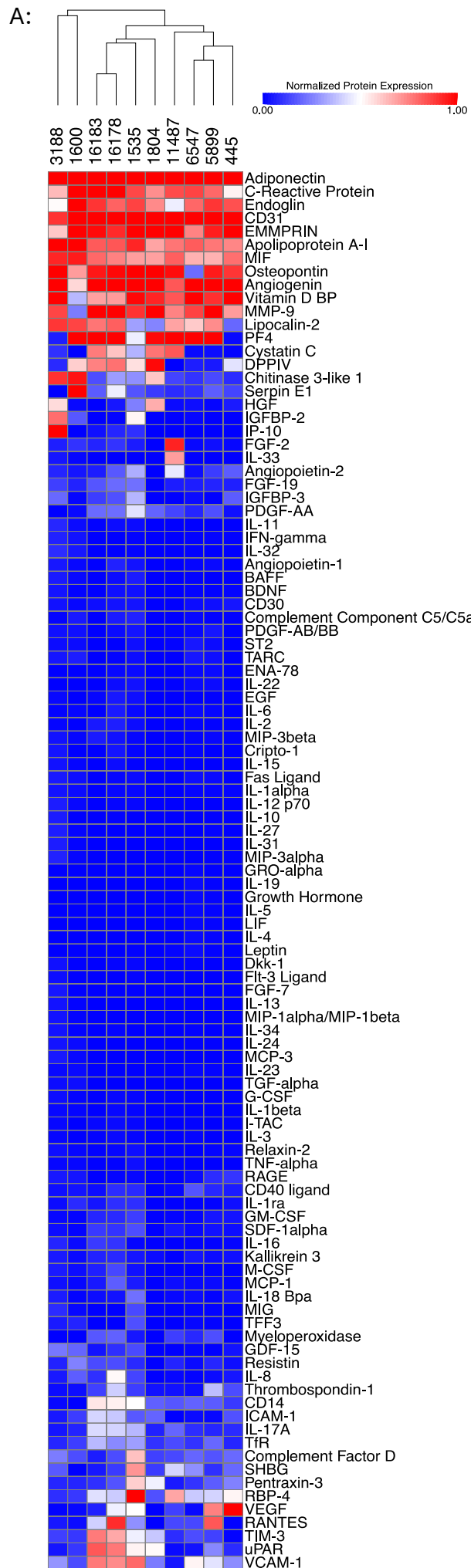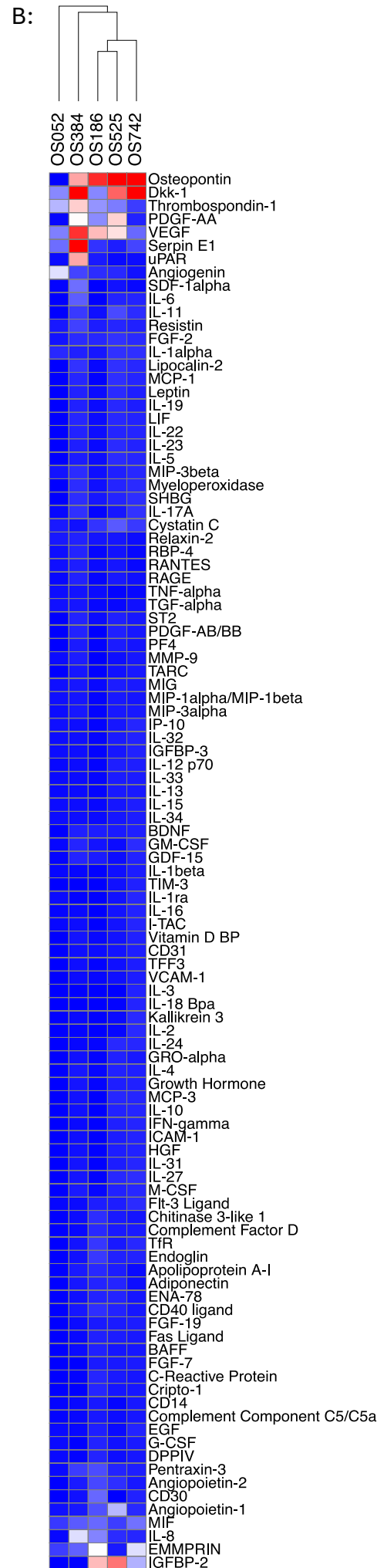

Supplemental Figure 2

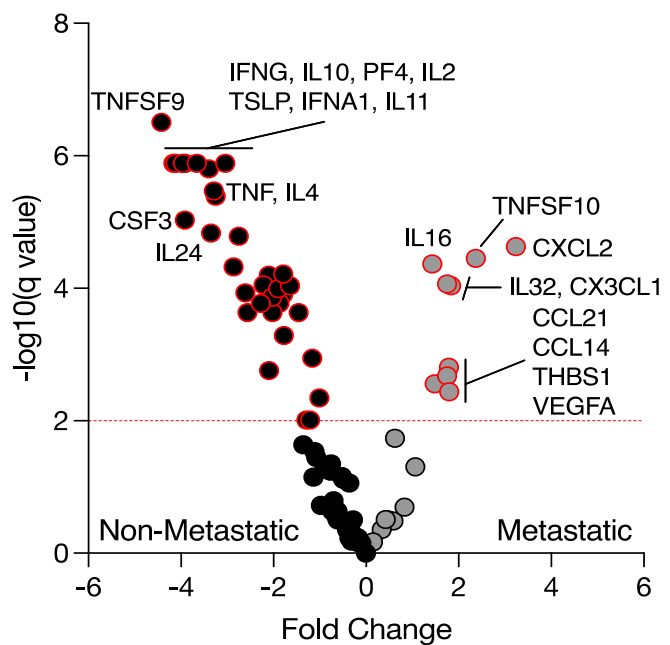

Supplemental Figure 3

Supplement: Supplement 3 — Supplemental Figure 1: Distribution and abundance of genes encoding functional and phenotypic macrophage markers. Clustered dot plots illustrating the spatial distribution of gene encoding different functional and phenotypic macrophage markers within each individual metastatic osteosarcoma specimen. The color bar above each dot plot corresponds to the different spatial clusters displayed on the adjoining spatial cluster maps that are shown for reference (above). Separate color palettes denote the independent clustering analysis of each specimen. Spatial clusters were localized to the extratumoral (black) or intratumoral (gray) microenvironments as depicted in the second color bar. Supplemental Figure 2: Chemokine/cytokine antibody array profiling in metastatic osteosarcoma patient specimens and patient derived cell lines. Heatmaps showing normalized protein expression of chemokines and cytokines from (A) metastatic osteosarcoma specimens and (B) patient derived cell line conditioned media. Supplemental Figure 3: Differential gene expression of chemokine/cytokines in primary versus metastatic osteosarcoma specimens. Volcano plot of differentially expressed chemokine and cytokine genes in non-metastatic (black dots) and pulmonary metastases (gray dots) from the Sorenson, et al. NanoString IO360 gene expression dataset.25 Horizontal red line indicates significance threshold (q = 0.001). Dots with red outline indicates q < 0.001. [file NIHPP2025.01.22.631350v1-supplement-3.pdf]
